# Combined Physical Exercise and Diet: Regulation of Gut Microbiota to Prevent and Treat of Metabolic Disease: A Review

**DOI:** 10.3390/nu14224774

**Published:** 2022-11-11

**Authors:** Li Zhang, Yuan Liu, Ying Sun, Xin Zhang

**Affiliations:** 1 Department of Physical Education, China University of Mining and Technology, Beijing 100083, China; 2 Department of Food Science and Engineering, Ningbo University, Ningbo 315211, China

**Keywords:** metabolic diseases, gut microbiota, diet, physical exercise

## Abstract

Background: Unhealthy diet and sedentary lifestyle have contributed to the rising incidence of metabolic diseases, which is also accompanied by the shifts of gut microbiota architecture. The gut microbiota is a complicated and volatile ecosystem and can be regulated by diet and physical exercise. Extensive research suggests that diet alongside physical exercise interventions exert beneficial effects on metabolic diseases by regulating gut microbiota, involving in the changes of the energy metabolism, immune regulation, and the microbial-derived metabolites. Objective: In this review, we present the latest evidence in the modulating role of diet and physical exercise in the gut microbiota and its relevance to metabolic diseases. We also summarize the research from animal and human studies on improving metabolic diseases through diet-plus-exercise interventions, and new targeted therapies that might provide a better understanding of the potential mechanisms. Methods: A systematic and comprehensive literature search was performed in PubMed/Medline and Web of Science in October 2022. The key terms used in the searches included “combined physical exercise and diet”, “physical exercise, diet and gut microbiota”, “physical exercise, diet and metabolic diseases” and “physical exercise, diet, gut microbiota and metabolic diseases”. Conclusions: Combined physical exercise and diet offer a more efficient approach for preventing metabolic diseases via the modification of gut microbiota, abating the burden related to longevity.

## 1. Introduction

Sedentary lifestyle has progressively become a habitual way of life in modern societies, and so contributes to the rising incidence of metabolic diseases such as Type 2 diabetes (T2D), obesity, cardiovascular diseases (CVD) and non-alcoholic fatty liver disease (NAFLD) [1]. Metabolic risks (namely high body mass index (BMI), high blood sugar, high blood pressure, and high cholesterol) accounted for nearly 20 % of total health loss worldwide in 2019, according to the World Health Organization (WHO) database. The Lancet published that high blood pressure contributed to one in five deaths (almost 11 million) in 2019, followed by high blood sugar (6.5 million deaths), high BMI (5 million), and high cholesterol (4.4 million). The costs associated with these diseases are enormous, but it has been estimated that these diseases are preventable by regular and adequate levels of physical exercise. Practically, evidence showing the benefits of regular physical exercise for health, regardless of age, has grown in recent years. Habitual exercise contributes to decreasing blood pressure and serum triglyceride levels, as well as improving high-density lipoprotein cholesterol levels, insulin sensitivity and glucose homeostasis [2]. The health-promoting mechanisms of physical exercise are complex and multifaceted, including better regulate immune–inflammatory responses, reductions in oxidative stress and adiposity, acceleration of the elimination of damaged mitochondria, and so on [3]. Intriguingly, a new factor by which exercise may affect metabolic diseases has emerged: the interplay with gut microbiota (GM) [4].

GM is engaged in various interplays affecting the health during the host’s entire life span. It acts as an endocrine organ, and the shifts of microbiota architecture and status promoted by exercise play an instrumental role in promoting the production of beneficial metabolites, stimulating/modulating the immune system, protecting the host from colonization of pathogens, and controlling lipid accumulation and insulin signaling [5]. In fact, positive effects have been reported, mainly in order to shape the diversity of microbiota, promote the formation of short-chain fatty acids (SCFAs), impact the integrity of the gut mucus layer, and maintain balance between beneficial and pathogenic bacterial communities [6]. Clinical research has revealed that α-diversity and SCFAs were increased and bacterial endotoxin lipopolysaccharide (LPS) was decreased in professional players than in non-athlete healthy subjects [7]. Meanwhile, regular exercise is a hormetic stressor to the gut that propels beneficial responses and improves the integrity of the intestinal barrier [8].

Diet is important in sculpting the microbial communities or metabolites in a manner that may affect disease [9]. The microbiota is exposed to healthy dietary components, such as dietary carbohydrates, proteins, vitamins, minerals and polyphenols, which can produce beneficial metabolites, in particular, SCFAs and tryptophan metabolites. These metabolites participate in the maintenance of intestinal mucosa integrity and also mediating host immune and homeostatic responses [6]. Conversely, an unhealthy diet, such as a high-fat diet (HFD), augments the production of pro-inflammatory cytokines, thereby leading to systemic chronic inflammation and LPS translocation, which increase the risk of metabolic diseases [4]. The effect of exercise on gut microbial composition or function is inextricably linked with dietary adjustments. The variety in the GM that seems to be associated with exercise may therefore be due to the combination with dietary intake, rather than exercise itself. According to the data from the WHO and The Centers for Disease Control and Prevention, regular physical exercise and dietary interventions can reduce the prevalence of gestational diabetes by 30% and the risk of death by 20% to 30% [1]. In this article, we review the research progress of the GM and its relationship with metabolic diseases, mainly including obesity, T2D, CVD, and NAFLD. We also focus on the effect of physical exercise, dietary components and dietary patterns on the GM. Importantly, this review presents some research and related mechanisms of preventing metabolic diseases by combining physical exercise and diet, which might provide a burgeoning avenue for the prevent of metabolic diseases.

## 2. Effect of Physical Exercise on Gut Microbiota

**Figure 1:**
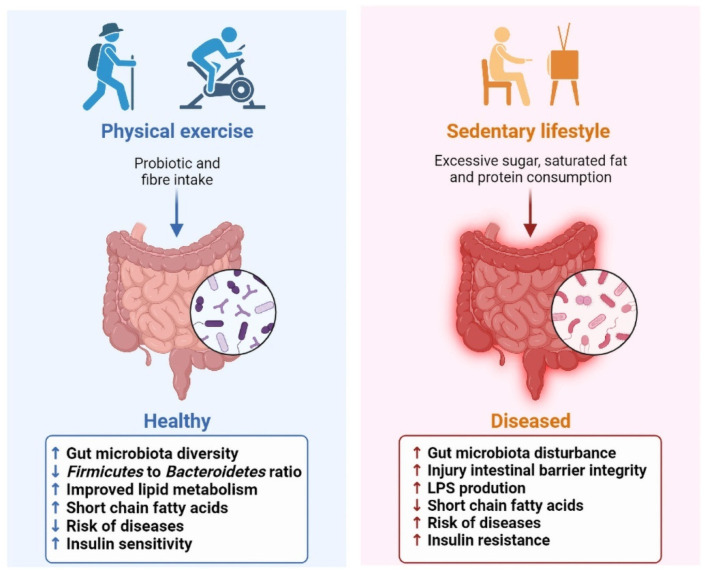
Effect of physical exercise on gut microbiota and host health.

Physical exercise is defined as a subset of physical activity that is planned, structured and repetitive and aims to either improve or maintain physical fitness [10]. Research demonstrates that regular exercise is performing physical exercise of moderate intensity for a minimum of 30 min, 5 days a week, or of high intensity for a minimum of 20 min, 3 days a week [10]. Habitual exercise suppresses the expression of basal pro-inflammatory cytokines, but excessive exercise triggers the production of multiple pro-inflammatory mediators. Reasonable and moderate physical exercise protects against all-cause mortality, and only in extreme cases can these adaptations contribute to an increased risk of physical exercise-associated complications [11]. In fact, regular exercise training independently effects gut function and microbiome characteristics, and then has a beneficial role in preventing metabolic diseases (Figure 1).

The role of physical exercise in shaping the diversity of the GM and modulating its distribution has been demonstrated. The changes in the GM, under exercise conditions, can affect the absorption of nutrients, and then affect host metabolism. Data from the American Gut Project indicated that adopting moderate exercise (from never to daily) reshaped the alterations in microbial composition and function, and promoted a healthier gut environment of elderly individuals, especially overweight elderly individuals [12]. The GM of professional rugby players exhibited greater α-diversity and a decrease in the *Firmicutes* to *Bacteroidetes* ratio [13]. Women performing the regular dose of exercise displayed a higher abundance of health-promoting taxa, such as *Faecalibacterium prausnitzii*, *Roseburia hominis* and *Akkermansia muciniphila*, compared to sedentary counterparts [14]. Research revealed that these microbes are known butyrate producers, having a beneficial effect on promoting intestinal barrier integrity, regulating the host immune system and lipid metabolism [15,16,17]. Similar results have been yielded in animals. Mice that performed physical exercise typically showed an increase in commensal taxa such as *Bifidobacterium*, *Lactobacilli* and *Akkermansia* [8,18]. Moderate exercise also alleviated chronic stress-induced intestinal barrier impairment in mice, reducing bacterial translocations and maintaining intestinal permeability [19]. Furthermore, significant quantities of lactate are released during exercise and then secreted into the gut lumen, which can alter intestinal pH [6].

Conversely, high-intensity exercise may be having a deleterious influence on intestinal function. A total of 70% of athletes might experience abdominal pain, nausea, and diarrhea after strenuous exercises [20]. Prolonged exercise also results in less microbial diversity, increases the abundance of *Helicobacter*, as well as induces an increased intestinal permeability, promoting bacterial and their toxic products to enter into the bloodstream and activate systemic inflammation [21]. Exhaustive and acute endurance exercise, as observed in animal studies, has been indicated to induce altered permeability [22].

## 3. Effect of Diet on Gut Microbiota

### 3.1. Nutrients

#### 3.1.1. Dietary Carbohydrates

A diet rich in different types and numbers of fruits, vegetables, and wholegrain cereals is the main sources of dietary carbohydrates (CHOs). In the human genome, less than 20 glycosidases have been identified as enzymes involved in digestion of dietary CHOs. Salivary α-amylase firstly breaks down complex CHOs into simple sugars in the mouth cavity, and digestible CHOs can be degraded and digested through pancreatic α-amylase, sucrase, maltase, galactose and lactase [23]. Complex non-digestible dietary CHOs drive our gut microbial to evolve an arsenal of carbohydrate-active enzymes in order to efficiently compete for nutrition [24].

The distal gut of the host is constantly inundated with a dynamic array of CHOs. It has been noted that simple CHOs (e.g., sucrose, fructose) cause rapid microbiota remodeling and hence metabolic disturbance in the host [25]. Complex CHOs, specifically, certain microbiota-accessible polysaccharides and dietary fiber, feed the dense consortium of microbes that compete in this habitat, having a major effect on gut microbial ecology and health [26]. A diet high in polysaccharides is related to up-regulated GM community diversity and promotes the growth of beneficial microbes, such as *Akkermansia*, *Bifidobacterium* and *Lactobacillus*. Meanwhile, GM can use intermediate oligosaccharides to generate host-beneficial SCFAs [27]. Pharmacological studies suggested *Dendrobium officinale* polysaccharides (DOPs) were indigestible and non-absorbing but promoted GM to produce more butyrate, mainly generated by *Parabacteroides*_sp_HGS0025, which mediated the improvement of intestinal health and immune function [28]. DOPs intervention also could reinforce the intestinal barrier function via promoting mucin synthesis, by acting on *Akkermansia muciniphila* [29]. Other polysaccharides from *Schisandra chinensis* also reversed the GM dysbiosis and upregulated the production of butyric acid and propionic acid, which is possibly involved in the anti-inflammation protective mechanism [30]. For dietary fiber, research indicated that an insulin-enriched diet reduced fasting blood glucose levels, as well as alleviated glucose intolerance and blood lipid panels in diabetic rats [31]. Remarkably, dietary fiber restriction not only contributes to a decrease in microbial diversity and the production of SCFAs, but also alters the metabolism of GM toward the utilization of less favorable substrates, which may be detrimental to the host [32].

#### 3.1.2. Dietary Proteins

Dietary protein is another key macronutrient, which also modulates microbial composition and metabolite production. The relationship between protein intake and health follows a U-shaped curve, in which a lower protein intake is associated with undernutrition states, while intake above the tolerable limit is associated with overnutrition illnesses [24]. WHO recommends a daily protein intake of 0.83 g/kg for adults [33]. The products of dietary protein digestion are amino acids. Metabolites of amino acids by GM degradation include SCFAs, branched chain fatty acids, indoles, phenols, thiols, sulfides, ammonia and amines [24]. On the one hand, protein degradation provides essential free amino acids as an alternative energy source for colonocytes [9]. On the other hand, this process also releases toxic metabolic by-products such as ammonia, sulfides and phenols, which are detrimental for the local intestinal environment [9]. Research showed that moderate dietary protein restriction could shape the harmonious balance of the microbiota composition and diversity, and improve gut barrier function in adult pigs [34]. Higher protein diets show a reduction in the abundance of CHO utilizers belonging to *Lachnospiraceae*, *Ruminococcaceae* and *Akkermansia* [35]. In addition, proteins, especially from red meat and processed meat, are a source of L-carnitine and choline, which can be metabolized by GM and produce trimethylamine (TMA) [36], subsequently oxidized to trimethylamine N-oxide (TMAO) [37]. High TMAO concentrations are correlated with an increased risk of CVD or death [38]. It is important to note that athletes may have a higher protein requirement to support bone metabolism, keep adequate protein synthesis and energy metabolism, as well as sufficient immune function and intestinal integrity in the intensive/prolonged exercise routines [39]. Research recommends that the protein intake of endurance- and strength-trained athletes was 1.2–1.7 g/kg/day [40]. Lack of protein, for instance, could lead to menstrual disorders in female athletes [41].

#### 3.1.3. Dietary Fats

Dietary fats from plants and animals are a reserve source of energy for the human growth and development. Fat is first digested by lingual and gastric lipases in the mouth. Dietary fat is hydrolyzed into free fatty acids (FFA) by pancreatic lipase; most of the FFA is absorbed in the small intestine, and a minority will pass through the gastrointestinal tract and directly alter GM composition [42]. A palm oil-based diet could induce body mass gains, negatively affect the microbiota diversity, and increase the ratio of *Firmicutes* to *Bacteroidetes*, compared to olive or safflower oil [43]. Regarding genera, saturated fatty acids decrease the abundance of *Bacteroides*, *Prevotella*, *Lactobacillus* spp. and *Bifidobacterium* spp. [44]. Consumption of HFD significantly also reduced the release of SCFAs compared with a low-fat diet [45]. The variation of the GM composition induced by dietary fats can also regulate the production of microbial-derived secondary bile acids (BAs). An HFD trigger enhanced BAs’ discharge, resulting in increased colonic concentrations of primary BAs. However, 5% to 10% of BAs are not reabsorbed but are converted to secondary BAs by microbes in the large intestine, which are harmful and promote colon carcinogenesis [46]. Moreover, the microbiota dysbiosis observed in HFD mice favored the passage of LPS from the intestinal lumen to systemic circulation, which activated the host pro-inflammatory signaling pathway and then triggered a low-grade systemic inflammation [9,47].

#### 3.1.4. Other Dietary Components

A stable gut microbial community is affected by several essential components, such as vitamins, minerals and polyphenols. Vitamins are required cofactors in small amounts for maintaining normal physiological function. Humans are incapable of synthesizing most vitamins to meet our daily needs, and they consequently have to be obtained exogenously. Remarkably, the GM has the capacity to regulate both the synthesis and metabolic output of various vitamins [24]. Subsequently, vitamins also can dramatically alter the abundance and diversity of the GM. Vitamin A, for example, can up-regulate the health-beneficial microbiota, including *Bifidobacterium*, *Lactobacillus* and *Akkermansia* genera [48]. Like vitamins, minerals are micronutrients that play an instrumental role for host metabolism and performing active interaction with the GM. It has been demonstrated that magnesium (Mg) deficiency is associated with an increased incidence of chronic disease [49] and reduces the *Bifidobacterial* content in Mg-deficient mice for four days [50]. While, with prolonged Mg deficiency (21 d), there is an increase in the abundance of *Bifidobacteria* and *Lactobacilli* [50]. Clinical trials are still necessary to identify the effects of magnesium deficiency and magnesium supplementation for avoiding adverse effects. In addition, polyphenols are a large and diverse family of compounds found widely in plant foods, several of which have been related to the gut health. Tea polyphenols could inhibit the growth of detrimental bacteria such as *Helicobacter pylori* and *Staphylococcus aureus*, and stimulate the growth or favor the growth of beneficial species of the GM, such as *Bifidobacterium* and *Akkermansia muciniphila* [51].

### 3.2. Dietary Patterns

It has been reported that dietary patterns may have a pronounced effect on the metabolic activity of the GM than individual nutrients. A single-nutrient dietary intervention has several limitations. Dietary habits worldwide are manifold, including the Western diet (WD), Mediterranean diet (MD), ketogenic diet (KD), intermittent fasting (IF) and so on (Table 1) [52]. In a WD, a large proportion of energy is provided by acellular nutrients, which are more easily digested by microbial and human cells [53]. Increased amounts of readily accessible acellular nutrients affect the regulation and maintenance of GM homeostasis by contributing to variations in pH, the GM composition and metabolism. On the other hand, the consumption of HFD also augmented the production of pro-inflammations cytokines, thereby leading to systemic chronic inflammation and LPS translocation [54]. As opposed to the WD, the MD is considered one of the most worldwide healthy dietary patterns. Greater adherence to the MD has been linked with a significant reduction in total mortality and reduces risk of immune system dysregulation, CVD, cognitive decline and cancer [55]. In addition, the MD changes the composition of the microbiota in favor of beneficial bacteria, such as *Parabacteroides distasonis*, *Bacteroides thetaiotaomicron*, and *Bifidobacterium adolescentis*, and counteracts the growth of pathogens, restoring potentially beneficial microbes [56]. The KD is a high-fat, adequate-protein, and low-CHOs diet. The body burns fats rather than CHOs to obtain calories by restricting the availability of CHOs. Research showed that the KD affected the GM with mixed results. On the one hand, the KD is at a greater risk of being nutritionally inadequate and may not maintain a healthy microbiota by lacking in fiber, necessary vitamins, minerals, and iron. On the other hand, research revealed that the KD conferred microbiota benefits and relieved colitis in a DSS-induced recipient, following the dramatic increase of the abundance of *Akkermansia* and butyric acid-producing *Roseburia*; additionally, the decrease of the abundance of *Escherichia/Shigella* was found in mice fed with a KD [57]. The IF is a dietary intervention similar to caloric restriction, encompassing various programs that manipulate meal time to improve body composition and overall health [58]. Overwhelming studies support the robust disease-modifying efficacy of the IF in animal models on a wide range of chronic disorders, including T2D, CVD, and brain function, in addition to weight loss [59]. The IF appears to have positive impacts on the GM. Preclinical studies consistently demonstrated the IF contributed to increasing the richness of gut microbes, enriching of the *Akkermansia muciniphila* and Lactobacillus, reducing putatively pro-inflammatory taxa *Desulfovibrio* and *Turicibacter*, and enhancing antioxidative microbial metabolic pathways [60].

## 4. Gut Microbial Dysbiosis Linked to Metabolic Diseases

Traditionally, genetic variants have been thought to be the major drivers of metabolic diseases, but the heritability of these variants is fairly modest. The GM is recently suspected to be a contributor for driving metabolic diseases. Compared with healthy individuals, most populations with obesity, T2D, CVD and NAFLD show reduced gut microbial diversity. The GM’s composition, if modified by external factors, leads to a dramatic change of the symbiotic relationship between GM and the host, which are essential for the development of metabolic diseases.

Alteration of the GM by behavioral changes, such as HFD and use of antibiotics, could be the robust drivers of the obesity pandemic. The researches concerning the role of the GM in mediating obesity pathogenesis, were based on findings from animal models firstly. The obese microbiota results in a significantly greater increase in harvesting energy from the diet. It has been observed that introduction of the microbiota from obese donors into germ-free (GF) mice results in an increased energy gain capacity, compared to those receiving the microbiota of lean donors [65]. Similarly, a transferrable obesity-associated microbiota contributes to the accumulation of total body fat than colonization with a ‘lean microbiota’ [66,67]. Following these phenomena, subsequent epidemiological studies have shown that GM composition differs between obese and lean individuals. [67]. Human studies observed that the microbiota of overweight individuals was characterized by a lower abundance of *Bacteroidetes* and a higher *Firmicutes* when compared with non-overweight individuals [68,69]. At the genus level, a metagenome-wide association study revealed the under-representation of *Bacteroides thetaiotaomicron* in obese individuals. Interestingly, gavage with *B. thetaiotaomicron* could alleviate diet-induced body weight gain and adiposity in mice, implying that probiotic or microbial compounds might be potential future modalities for anti-obesity [70].

T2D has also been considered to be under the influence of the dysregulated GM composition and functionality. Clinical reports have indicated the relative abundance of *Bifidobacterium*, *Lactobacillus* and butyrate-producing bacteria (*Akkermansia muciniphila*) were negatively associated with T2D, while the genera of *Clostridium* spp., *Ruminococcus*, *Fusobacterium* and *Blautia* were positively associated with T2D [71,72]. The dysregulation of the GM may impair intestinal barriers through damaging tight junction proteins (TJPs), subsequently causing a leaky mucosa and metabolic endotoxemia, which is one of the leading factors in insulin resistance and the development of T2D [73]. In addition, indirect evidence that the GM might be involved in glucose regulation comes from large-scale epidemiological studies, which revealed that patients with total colectomy had an increased risk of T2D compared with those without colectomy [74].

There are many pathological processes and risk factors of CVDs involved in obesity, T2D, dyslipidemia, hypertension, and an unhealthy lifestyle, such as partaking in smoking, lack of exercise and poor dietary habits [75]. Noteworthy, most of those factors are associated with the GM, and genome sequencing and metagenomic analyses also revealed the association between CVD phenotypes and changes of specific microbial taxa, or the GM richness and diversity. Early study demonstrated that bacterial DNA (mainly of *Chryseomonas*) was detected in atherosclerotic plaques with signatures that match taxa associated with disease states [76]. Moreover, a metagenomic analysis showed that the gut microbiome of CVD patients differed from those of healthy individuals, which was mainly manifested in elevated abundances of *Streptococcus* spp. and *Enterobacteriaceae* spp., and in the decreased abundances of *Bacteroides* spp., *Prevotella copri*, and *Alistipes shahii* [77,78]. At the mechanistic level, the effect of the GM on CVDs has been linked to modulation of inflammation, intestinal barrier function and metabolites. Dysbiosis-associated changes in the GM impair intestinal barriers, leading to an elevation in circulating LPS levels, and LPS can activate inflammatory signals through the toll-like receptor (TLR)-MyD88 signaling pathway, resulting in the release of pro-inflammatory cytokines that orchestrate an inflammatory state in the host [79]. Previous studies showed patients with heart failure displayed impaired intestinal integrity, and that elevated levels of pro-inflammatory cytokines in the blood are associated with symptom severity and poorer outcomes [80]. In metabolism-dependent pathways, GM cleaves some TMA-containing compounds to produce TMA, which can be further oxidized to TMAO by flavin monooxygenase. TMAO activates MAPK, NF-κB signaling pathways, contributing to inflammatory gene expression, which affects lipid metabolism and increases triglycerides, and decreases high-density lipoproteins in CVD patients [81].

NAFLD is a disorder associated with obesity, generally regarded as the hepatic manifestation of the metabolic syndrome. Multiple preclinical and clinical studies have highlighted a role of the GM in NAFLD pathogenesis, although we are still far from finding a causal link. In brief, individuals with NAFLDs harbor lower GM diversity than healthy subjects, having an increased abundance of species assigned to *Anaerobacter*, *Streptococcus*, *Escherichia* and *Lactobacillus*, and a lesser abundance of *Prevotella*, *Oscillibacter* and *Alistipes* spp [82,83,84]. The mechanism by which GM is proposed to affect NAFLD is in terms of the gut–liver axis. Aside from dysregulation of the GM, NAFLD is also related to the enterohepatic circulation of bile acids, GM-mediated inflammation of the intestinal mucosa and the related impairment in mucosal immune function [52].

## 5. Combined Physical Exercise and Diet for Preventing Metabolic Diseases by Modulating Gut Microbiota

Consumption of a calorie-rich diet and sedentary lifestyle have contributed to the rising incidence of obesity in the modern lifestyle, which is caused by energy intake exceeding energy expenditure to a large extent. Substantial epidemiologic evidence suggests obesity is a risk factor for inducing other metabolic diseases, including T2D, CVD and NAFLD. Identifying effective interventions is an important way for improving metabolic diseases. In fact, the majority of research concluded that when a program includes diet alongside physical exercise, there were more effective changes, compared with exercise or diet alone [85]. The diversity and function of GM are also affected by diet and physical exercise. Here, we will summarize the research from animal and human and potential mechanisms on improving metabolic diseases through diet-plus-exercise interventions.

### 5.1. Evidence from Animal Studies

The effects of exercise and diet on GM are more extensively focused on in HFD animal models. Repeated exercise increased the α-diversity and metabolic capacity of the mouse distal GM during diet-induced obesity [86]. Moderate exercise and a low-fat diet have beneficial effects on body weight loss and macrophage immunocompetence in HFD-induced obese mice [4]. Exercise plus curcumin in combination exhibited better effective in weight loss and improved glucose homeostasis and lipid profiles of diabetic rats, compared with exercise or diet alone interventions groups [87]. Besides, the combined treatment of isoflavones and exercise has a stronger impact on enhancing GM diversity and preventing HFD-induced inflammation [88].

### 5.2. Evidence from Human Studies

Although diet-plus-exercise interventions are classically accepted, few human studies deeply reveal the effect of the combination of physical exercise and diet on GM and metabolic diseases, which often focus on meta-analysis studies. A meta-analysis from Johns et al. identified that there was no difference in weight loss in the short-term for diet-only/exercise-only interventions than for combined physical exercise and diet, but in both the short and long term, weight had a greater reduction in the diet-plus-exercise interventions groups [36]. Wu et al. conducted a meta-analysis of weight loss studies published from 1997 to 2008. Results indicated that the weighted mean difference for the 1–2 year time point between combined physical exercise groups and diet-only controls was −2.29 vs. −0.67 kg/m^2^ for BMI, respectively, implying that diet-plus-exercise interventions yielded a more long-term weight loss effect than diet-only interventions [89]. The diet-plus-exercise interventions was also found to be superior in improving the body weight and adiposity of overweight/obese postmenopausal women, compared with diet-only interventions [90]. Moreover, a 6 month randomized intervention program suggested that aerobic exercise and a low-CHO diet offer a more efficient approach for reducing liver fat and preventing diabetes via modification of GM composition [91]. A randomized controlled trial for overweight/obese Chinese females (BMI 25.1 ± 3.1 kg/m^2^) revealed that a combined low-carbohydrate diet with exercise training increased the SCFAs-producing *Blautia* genus and reduced T2D-related genus *Alistipes*, caused significant weight loss, as well as improved blood pressure, insulin sensitivity and cardiorespiratory fitness, suggesting that a low-CHO diet and exercise interventions might play a role in cardiometabolic health by regulating the GM [92,93]. A recent randomized controlled trial demonstrated that diet-plus-exercise interventions could significantly reduce hepatic fat content and increase the diversity and stabilize of keystone microbes than exercise or diet alone interventions, which offered a more efficient avenue for developing diet-plus-exercise intervention strategies for preventing NAFLD [94].

### 5.3. Underlying Mechanisms

**Figure 2:**
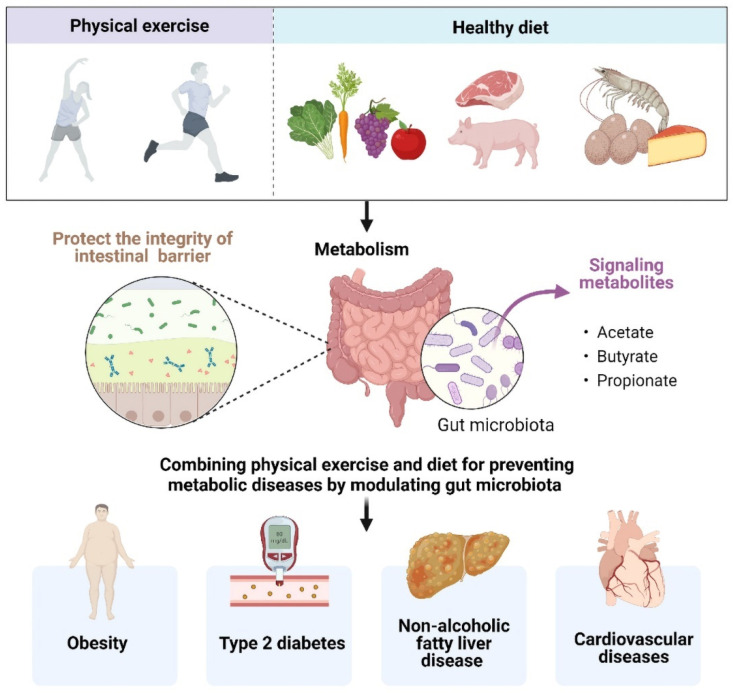
Combining physical exercise and diet for preventing metabolic diseases by modulating gut microbiota.

The key is to understand the potential mechanism that a combined diet and exercise strategy may prevent metabolic diseases (Figure 2). Several studies have elucidated that exercise in the fasted state generated advantageous metabolic adaptations, accompanying by stable blood glucose concentrations and elevated blood FFA concentrations, which may be more effective in improving insulin sensitivity and controlling glycemic in insulin-resistant individuals [95,96]. From the GM perspective, combining physical exercise and diet tempers intestinal barrier dysfunction, reserving mucous thickness and intestinal permeability. The intestinal barrier is a selective physical and immunological barrier that facilitates nutrient, water, and electrolyte absorption into circulation while deterring the translocation of harmful pathogens and noxious luminal substances [97]. As previously mentioned, one of the pathophysiological statuses by which metabolic diseases could be perpetuated and aggravated was intestinal homeostasis dysbiosis to release endotoxins, creating a leaky gut, which induces a chronic low-grade inflammatory state in the host [98]. Diet and exercise can modulate the expression of TJPs involved in the maintenance of epithelial membrane integrity, which improves intestinal permeability and reduces the risk for chronic disease [99].

The combination of diet and exercise also can influence how the GM utilizes and synthesizes metabolites. The GM and the corresponding metabolites act in concert with the host in different ways, affecting intestinal homoeostasis and providing protective intervention for metabolic diseases. Specifically, SCFAs are one of the major end products of microbial fermentation or the transformation of dietary polysaccharides in the gut. Exercise is a potent modulator of SCFAs, exerting a particular influence on butyrate concentrations [16]. SCFAs are the primary energy source for the intestinal epithelial cells, participating in the maintenance of intestinal mucosa integrity, which also improves glucose and lipid metabolism, controls energy expenditure as well as regulates the immune system and inflammatory responses [100]. In animal models, the supplementation with SCFAs has been shown to improve the metabolic phenotype by increasing energy expenditure and glucose tolerance, and might help delay or attenuate diabetes and lead to weight reduction [101].

## 6. Conclusions and Future Perspectives

Regular and adequate levels of physical exercise and diet interventions abate the burden related to longevity and expand life expectancy of present days. Regular exercise is a hormetic stressor to the gut that propels beneficial responses, particularly in shaping the diversity of the GM and modulating its distribution. Healthy dietary components and patterns combined with physical exercise propels the production of beneficial metabolites and tempers intestinal barrier dysfunction, which protects the host against invading microorganisms, contributing to maintaining homeostasis and preventing metabolic diseases. However, additional challenges and limitations in the area of research are numerous. Although both interventions are traditionally accepted and implemented, few in-depth studies focus on the mechanism of microbiota-based strategies coupled with physical exercise programs to delay metabolic disease onset. More research is needed to determine whether the GM could be an important predictor of metabolic diseases in response to dietary and exercise interventions. Exercise intensity is a controversial issue; we must be take into consideration the various forms of exercise, and the exercise duration. Meanwhile, we should formulate different intervention plans according to different populations; the challenge is how to motivate the sedentary people to escape from unhealthy lifestyles.

## Figures and Tables

**Table 1:** The effect of dietary patterns on health mediated by gut microbiota.

Dietary Pattern	Characteristic	Changes of Gut Microbiota	Effect	Reference
WD	High consumption of saturated and trans fatty acids, refined grains, sugar, salt, alcohol and other harmful elements; Low content of complex dietary fiber.	*Firmicutes*/*Bacteroidetes* ratio↑ *Alistipes*↑ *Bilophila*↑ *Bifidobacteria*↓	Systemic chronic inflammation and LPS translocation; Increase the risk of disease.	[61]
MD	High intake of whole grains and vegetables; Use olive oil as the lipid supply; A regular but moderate consumption of fish and other meat, dairy products and red wine.	*Bifidobacteria*↑ *Lactobacillus*↑ *Clostridium*↑ *Faecalibacterium*↑ *Oscillospira*↑ *Ruminococcus*↓ *Coprococcus*↓	Improve the gut barrier integrity; Protect against oxidative stress and inflammation; Reduce the total mortality and the risk of cardiovascular, metabolic and gastrointestinal diseases.	[56]
KD	High-fat, adequate-protein, and low-carbohydrate.	*Akkermansia*↑ *Parabacteroides*↑ *Escherichia*↓ *Shigella*↓	Nutritionally inadequate in fiber, necessary vitamins, minerals, and iron.	[57]
IF	Manipulate meal time to improve body composition and overall health, including of time-restricted feeding, alternate day fasting, and religious fasting.	*Akkermansia*↑ *Lactobacillus*↑ *Desulfovibrio*↓ *Turicibacter*↓	Improve gut epithelial integrity, the leaking LPS and blunted systemic inflammation; Improve metabolic profiles and reduce the risk of obesity, obesity-related conditions.	[62]
VD	Reduce or restrict of animal-derived foods; High intake of plant-source foods.	*Bacteroides*/*Prevotella* ratio↑ *Clostridium*↑ *Faecalibacterium*↑ *Bifidobacteria*↓	Reduce of caloric intake but nutritional deficiency of fatty acids, proteins, vitamins, and minerals; Prevent and better control of chronic diseases.	[63]
GD	The exclusion of gluten-containing cereals like wheat, rye, barley and hybrids.	*Bifidobacterium* ↓ *Lactobacillus*↓ *Enterobacteriaceae*↑ *Escherichia coli*↑	Appropriate for treatment of celiac disease, dermatitis herpetiformis and gluten ataxia.	[64]

WD = Western diet, MD = Mediterranean diet, KD = ketogenic diet, IF = intermittent fasting, VD = vegetarian diet, GD = gluten-free diet, ↑ = up-regulate, ↓ = down-regulate.
